# Editorial: The impact of advancing global oncology: towards equity in precision prevention and medicine

**DOI:** 10.3389/fonc.2026.1829973

**Published:** 2026-04-10

**Authors:** Jaira Ferreira de Vasconcellos, Juliano D. Paccez, Khadijah A. Mitchell

**Affiliations:** 1 Department of Biology, James Madison University, Harrisonburg, VA, United States; 2 Center for Translational Research in Oncology (LIM24), Instituto do Câncer do Estado de São Paulo (ICESP), Hospital das Clínicas da Faculdade de Medicina da Universidade de São Paulo (HCFMUSP), São Paulo, SP, Brazil; 3 Comprehensive Center for Precision Oncology, Universidade de São Paulo, São Paulo, SP, Brazil; 4 Cancer Prevention and Control Program, Fox Chase Cancer Center, Temple University Health System, Philadelphia, PA, United States

**Keywords:** cancer, global oncology, health disparities, health equity, precision medicine, precision prevention

Precision prevention tailors cancer risk reduction strategies by integrating biological, environmental, behavioral, and socioeconomic factors that shape disease risk (1). As highlighted in this Research Topic, these can include both non-modifiable (genetics and nulliparity) and modifiable risk factors (tobacco smoking, alcohol consumption, obesity, and social determinants of health). Precision medicine tailors healthcare delivery by integrating biological, environmental, and lifestyle data to prevent, diagnose, and treat cancer in individuals and populations (1, 2). As outlined below, a variety of cancer types have benefited from advances in precision oncology research and practice, including hereditary cancers (BRCA-associated), common somatic cancers (prostate, colorectal, gastrointestinal, and pancreatic), and rare somatic cancers (uterine carcinosarcoma). As the global cancer burden continues to rise, integrating individual and population-level risk factors at various geographic and environmental scales with molecular and sociodemographic data is essential for promoting equity in existing and emerging (artificial intelligence in oncology) strategies. This would ensure the benefits of precision prevention and medicine extend to individuals and populations worldwide. Several studies in this Research Topic illustrate how these principles are applied to understand cancer burden and risk across populations.

Global, regional, and national disease burden were investigated in two original research articles. Liu and Jiang investigated the prostate cancer burden, showing that the global incidence of prostate cancer progressed from ~0.51 million cases in 1990 to ~1.32 million cases in 2021. Declines were observed in the global estimated annual percentage change of age-standardized (i) incidence rates, (ii) disability-adjusted life years, and (iii) mortality rates, while an increase was observed in age-standardized prevalence rates. Low to middle-socio-demographic index countries showed the most pronounced rate increases, with men over 50 years the most affected. Overall, burden metrics continue to increase in low to middle-socio-demographic index quintile countries. Simultaneously, only modest improvements in age-standardized disability-adjusted life years and mortality were observed in regions with high socio-demographic index, demonstrating that prostate cancer continues to be a significant public health challenge worldwide. Deng et al. investigated the colorectal cancer burden and demonstrated that approximately one-third of all colorectal cancer-related deaths were attributable to smoking, alcohol consumption, and obesity. The age-standardized death rate from colorectal cancer associated with smoking and drinking is significantly increased in low, low-to-middle, and middle social demographic index regions. Men show a disproportionately high burden of smoking-related colorectal cancer mortality. Overall, these findings may be critical towards the development of interventions and/or public health policies to reduce the disease burden of colorectal cancer worldwide. In a brief research report, Ramirez Leon et al. described the use of family history (FH) screening services for BRCA-associated cancers among Latinas in Georgia. The FH screening was an effective tool, demonstrating the importance of approaches to equitable cancer genetic screening dissemination. More broadly, these efforts could inform public health policies and strategies for tailoring and implementing successful population-based cancer genetic FH screening across different areas. Finally, in a case report, Sun et al. described the case of a woman who had never given birth (nulliparous) and was diagnosed with uterine carcinosarcoma. They also reviewed the literature and found that 15.47% of women diagnosed with uterine carcinosarcoma were nulliparous. This study informs the field in this rare and aggressive form of uterine carcinosarcoma and highlights the high rate of nulliparous patients reported to develop the disease. Beyond these original studies, several systematic reviews further explored the factors shaping cancer disparities worldwide.

The systematic reviews presented in this Research Topic offer valuable insight into the multiple dimensions that shape cancer disparities, including the influence of new technologies, social conditions, and preventable risk factors. Together, they illustrate how these elements interact to affect cancer outcomes across different populations and healthcare settings. Artificial intelligence (AI) is increasingly becoming part of routine oncology research and clinical practice. In their study, Xu et al. document a clear expansion in AI-related publications in oncology over the last 30 years, with particularly strong growth in the USA and China. Much of this work has focused on developing models to estimate cancer risk, support symptom monitoring, and assist clinical decision-making. These developments underscore the pivotal role of AI in supporting more individualized approaches to cancer prevention and management. Simultaneously, the concentration of research in a limited number of regions highlights the importance of expanding access to these technologies more broadly. In parallel, social determinants of health continue to be strongly associated with cancer prognosis. Santellano et al. reported that survival among patients with gastrointestinal malignancies is closely linked to socioeconomic conditions. Individuals without health insurance, with lower income, or without family support had poorer outcomes. These observations reinforce the reality that cancer survival is shaped not only by biological factors and treatment, but also by access to care and social support. They also point to the need for more consistent and prospective studies to better understand how these factors can be addressed in clinical practice and public health policies. Additionally, modifiable behavioral exposures remain another key contributor to cancer burden. Du et al. examined the impact of smoking on pancreatic cancer worldwide and found that, although some declines have occurred, the overall burden remains substantial. Men and older individuals continue to be the most affected, and trends suggest a growing impact in regions such as East Asia. These findings highlight the continued importance of tobacco control, along with efforts to improve early detection and prevention, as essential components of strategies aimed at reducing cancer incidence and mortality.

Together, the contributions in this Research Topic highlight the worldwide burden of different types of cancer and cancer disparities and provide critical insights into how population-based FH screening services may positively contribute to public health policies and how AI development may support novel and targeted approaches to cancer prevention and management. Continued research efforts are needed to tailor prevention and treatment efforts and to translate current research findings into global subpopulations. We look forward to the continued advancement of global oncology and to the day when equity in precision prevention and medicine is a reality worldwide.

## References

[1] Bíró K Dombrádi V Jani A Boruzs K Gray M . Creating a common language: defining individualized, personalized and precision prevention in public health. J Public Health (Oxf). (2018) 40:e552–9. doi: 10.1093/pubmed/fdy066, PMID: 29897560

[2] Collins FS Varmus H . A new initiative on precision medicine. N Engl J Med. (2015) 372:793–5. doi: 10.1056/NEJMp1500523, PMID: 25635347 PMC5101938

